# Urticaria and HIV Infection: A Case Report

**DOI:** 10.7759/cureus.29662

**Published:** 2022-09-27

**Authors:** Anaahat K Brar, Jackline Grace

**Affiliations:** 1 Family Medicine, San Joaquin General Hospital, French Camp, USA

**Keywords:** chronic urticaria, infections, skin rash, hiv, acute urticaria

## Abstract

Urticaria is a common skin rash, which can be acute or chronic. It can be idiopathic or associated with various disorders and infections, but rarely human immunodeficiency virus (HIV). We report a case of an adult male presenting with a new onset of chronic urticaria. Its workup revealed newly diagnosed HIV. The frequency and severity of urticaria wheals improved significantly with HIV treatment.

## Introduction

Acute and chronic urticaria have a lifetime prevalence of 20% and 5%, respectively [1-2]. It involves wheals (hives), angioedema, or both [1]. A wheal is a superficial, erythematous, itchy skin lesion with eventual central clearing, and it can last from a few minutes up to 24 h. The lesion size varies from a few millimeters to several centimeters and involves superficial layers of skin, mainly on the trunk and proximal parts of limbs. Angioedema involves a sudden onset of non-pruritic, burning or painful, skin-colored, or erythematous swelling of deeper layers of skin/subcutaneous tissue (mainly around eyes and mouth) or mucous membranes. Urticaria involves wheals alone in 40% of cases, angioedema alone in 11% of cases, and both in 49% of cases [3]. Urticaria affects patients’ quality of life significantly. Its pathophysiology involves mast cell activation that releases histamine and other chemicals like cytokines, which in turn results in vasodilation, plasma leakage or swelling, and sensory nerve ending stimulation [1]. 

Zuberbier et al. [1] noted that finding the individual trigger for these mast cell activations and subsequent releases can be challenging, especially in chronic cases. Its work up includes a detailed history, physical examination, lab tests: complete blood count (CBC), erythrocyte sedimentation rate (ESR), C-reactive protein (CRP), total immunoglobulin E (IgE), and specific or provocative tests, only if a specific trigger is suspected. Once the trigger is identified, it should be avoided, and H1 antihistamines are recommended as first-line therapy. Refractory cases may require higher doses of antihistamines, corticosteroids, omalizumab, or ciclosporin. Differential diagnoses of urticaria include urticaria vasculitis, exercise-induced anaphylaxis, other mast cell disorders, Still’s disease, Well’s syndrome, and Schnitzler’s syndrome.

Zuberbier et al. [1] subdivided urticaria based on duration: acute (less than six weeks) or chronic (repeated lesions lasting more than six weeks). Based on etiology, it is subdivided into spontaneous or inducible with underlying triggers (dermographism, cold, heat, delayed pressure, solar, vibration, cholinergic, contact, or aquagenic). Radonjic-Hoesli et al. [2] mentioned that chronic spontaneous urticaria might have a loose association with some triggers because their presence does not always induce symptoms. These triggers could be stress, obesity, food (nuts, chocolate, eggs, shellfish, preservatives), or drugs (non-steroidal anti-inflammatory drugs, antibiotics, and contrast agents). Ronit et al. [4] noted that these patients are more likely to have other autoimmune diseases, especially hypothyroidism, hyperthyroidism, and rheumatoid arthritis. Type 1 diabetes, Sjögren syndrome, celiac disease, and systemic lupus erythematosus are also more prevalent in these patients. Urticaria and autoimmune diseases are both more common in women. Chronic spontaneous urticaria also occurs in patients with connective tissue diseases, autoimmune diseases, malignancies, and infections [3-4]. These infections are caused by viruses (herpes simplex 1 and 2, human herpesvirus 6, cytomegalovirus, Epstein-Barr virus, hepatitis A, B, and C viruses) [5]. There are case reports of urticaria in human immunodeficiency virus (HIV) patients; these patients have a high incidence of other cutaneous lesions and disorders [6-11]. Urticaria also occurs in patients with coronavirus disease 2019 (COVID-19), infestation with parasites, bacterial infections (Helicobacter pylori and bacterial infections of nasopharynx), and rarely helminths [1, 12-16].

## Case presentation

A 28-year-old male presented to the clinic with recurrent, intermittent itchy rashes on his chest for three weeks. The rash consisted of multiple small, less than 1 cm, discrete, circular lesions. The rash would disappear spontaneously within 24 h and reappear in different areas of the skin. He was evaluated in the emergency department twice prior to his visit and was diagnosed with urticaria/hives. The hives would resolve using diphenhydramine on an as-needed basis, but their frequency increased to daily. He did not present with hives at the time of the initial clinic visit, but hives were confirmed from a picture taken by the patient. 

The patient denied any symptoms of angioedema, difficulty breathing, throat swelling, choking, or coughing. He had a perianal abscess and fistulotomy six weeks prior to the appointment, but he was asymptomatic at the time of the visit; his only medications were docusate sodium and psyllium. He denied recent changes in diet, exposure to pets or chemicals, soap/detergent/cologne use, new bedding, or clothes. There was no history of rash on palms or soles, weight loss, fever, night sweats, sore throat, genital rash or discharge, joint pain, sensitivity to cold or heat, recent stress, abdominal pain, diarrhea, tiredness, or lymphadenopathy. He denied a history of asthma, atopic dermatitis, photosensitivity, seasonal allergies, food or nut allergies, insect stings, thyroid disease, Raynaud’s, or sexually transmitted infections (STIs). Screening tests for gonorrhea, chlamydia, syphilis, HIV, and hepatitis were negative one year ago. He is heterosexual, sexually active with one female partner with intermittent condom use in the past one year. He declined to elaborate on his previous sexual history, but he denied having male sexual partners. There was no history of tobacco, marijuana, alcohol, or illicit drug use. 

On examination, he was a healthy-looking male with no distress, a height of 165 cm, weight of 61.7 kg, body mass index (BMI) of 22.66 kg/m², afebrile with a temperature of 36.3℃, heart rate of 64 beats per minute, respiratory rate of 14 breaths per minute, and blood pressure 135/81 mmHg. Normal skin examination, including perianal area. No neck or groin lymphadenopathy. Normal breath sounds, heart sounds, and abdominal examination. 

He was diagnosed with urticaria and instructed to continue diphenhydramine as needed for itching and cetirizine daily until the next follow-up. Additional blood tests included: ***complete metabolic panel (CMP), rapid plasma reagin (RPR), acute hepatitis panel, thyroid stimulating hormone (TSH), COVID-19, and CBC, which were all within normal limits, except for fluctuating white blood cell (WBC) count (2500, 3000, 4400, 1900). Thus, an HIV test was ordered after counseling. HIV screening and confirmatory tests were positive. Chlamydia and gonorrhea tests were negative. At the time of diagnosis, lab values were: CD4 count 83 cells/mm3 and HIV viral load 649,961 copies/mL. The patient started Biktarvy (bictegravir, emtricitabine, and tenofovir alafenamide) and Bactrim DS (trimethoprim/sulfamethoxazole). 

After approximately one month of treatment for newly diagnosed HIV/AIDS, the patient reported feeling well overall. Originally he had hives daily, after one month of HIV treatment it was reduced to a few times a week, and after two months of HIV treatment, it disappeared completely. He continued to have no respiratory symptoms. His subsequent lab work showed improvement (Table 1). 

**Table 1:** Initial and post-treatment laboratory results.

Time of labs	HIV viral load	CD4 count
Initial	649,961 copies/mL	83 cells/mm3
One month follow up	Below 30 copies/mL	172 cells/mm3
Two month follow up	Below 30 copies/mL	266 cells/mm3

## Discussion

Although HIV patients have a high prevalence of skin disorders [11, 17-20], there are only a few case reports of urticaria in these patients. The skin immune system involves a high density of dendritic cells and Langerhans cells with receptors, which are targets for HIV [19-20]. Skin manifestations in HIV patients are subdivided into primary (an unexplained mechanism) and secondary (caused by decreased dendrites, macrophages, and natural killer cells as the disease progresses) [19]. Primary lesions include seborrheic dermatitis, xerosis, atopic dermatitis, psoriasis, drug-induced, and HIV-related pruritus. Secondary manifestations are caused by infections (Herpes simplex, Varicella-Zoster, Human Papillomavirus, bacteria, Candida, dermatophytes), or neoplasms (squamous cell carcinoma, basal cell carcinoma, Kaposi’s sarcoma, T-cell lymphoma) [19].

Our patient had chronic spontaneous urticaria as the presentation of his hives persisted for at least six weeks. The etiology of the hives in this patient was most likely HIV because the urticarial symptoms decreased from daily to a few times a week after one month of HIV treatment and completely disappeared after two months of HIV treatment. This was evident by the improving CD4 counts and the declining HIV viral load. Antibiotics and stress from recent perianal abscess/fistulotomy were less likely the triggers because urticarial rash started weeks after the abscess resolution. Differential diagnosis of urticaria in this newly diagnosed HIV patient could also be acute retroviral syndrome, which can cause fever, chills, fatigue, sore throat, muscle aches, and rash [11]. A rash caused by acute HIV infection is typically maculopapular, red or purple, depending on skin color, and persists for days, unlike hives in this patient. Another possibility could be eosinophilic folliculitis, which involves itchy urticarial papules and pustules on upper body parts. Eosinophilic folliculitis rash is persistent compared to the fleeting nature of urticaria, and it occurs in the late stages of AIDS [18]. The papular pruritic eruption is less likely because of the lack of firm papules on the trunk, face, and extremities. Other viral infections are unlikely because they cause persistent vesicles [18]. 

Our hypothesis that HIV was a trigger for chronic urticaria in our patient was supported by other case reports [6-10]. Lin and Schwartz [9] reported three cases of cold urticaria in HIV patients and that could be from the altered immune system, high IgE levels, and hypersensitivity phenomenon in these patients. Similarly, Yu et al. [6] reported one case of cold urticaria in an HIV patient with extremely low CD4 cell count and high IgE levels. They highlighted the inverse relationship between IgE and CD4 cell count. 

In addition to hypersensitivity, HIV has reportedly been associated with an allergic component. Reche et al. [7] reported one case of transient acquired C1 inhibitor functional deficiency and angioedema in an HIV patient. Angioedema symptoms disappeared with HIV treatment. Imbalzano et al. [5] noted that urticaria is associated less with allergic diseases, and more often with systemic diseases, especially viral infections. They hypothesized that viruses act as potential triggers or main etiologic agents in causing urticaria, likely through cross-reactivity between virus antibodies and circulating immune complexes with host cells. Wedi et al. [13] noted a link between viral infections and urticaria, but it is difficult to test because patients cannot be rechallenged (it is unethical to infect a healthy subject with an infection to reproduce urticaria).

## Conclusions

This case report and supportive literature show HIV as a potential trigger for urticaria development. This patient’s urticaria symptoms improved significantly with HIV treatment, as demonstrated by an improved CD4 count and decreased HIV viral load. The underlying mechanism of association between HIV and urticaria is not known. It may be related to HIV receptors on dendritic cells, making them targets for HIV. Direct association between the two diseases cannot be proven because of a lack of rechallenge possibility.

## References

[1] Zuberbier T, Abdul Latiff AH, Abuzakouk M (2022). The international EAACI/GA²LEN/EuroGuiDerm/APAAACI guideline for the definition, classification, diagnosis, and management of urticaria. Allergy.

[2] Radonjic-Hoesli S, Hofmeier KS, Micaletto S, Schmid-Grendelmeier P, Bircher A, Simon D (2018). Urticaria and angioedema: an update on classification and pathogenesis. Clin Rev Allergy Immunol.

[3] Bonner JR (1990). Urticaria and angioedema. Clinical Methods: The History, Physical, and Laboratory Examinations, 3rd ed.

[4] Confino-Cohen R, Chodick G, Shalev V, Leshno M, Kimhi O, Goldberg A (2012). Chronic urticaria and autoimmunity: associations found in a large population study. J Allergy Clin Immunol.

[5] Imbalzano E, Casciaro M, Quartuccio S, Minciullo PL, Cascio A, Calapai G, Gangemi S (2016). Association between urticaria and virus infections: a systematic review. Allergy Asthma Proc.

[6] Yu RC, Evans B, Cream JJ (1995). Cold urticaria, raised IgE and HIV infection. J R Soc Med.

[7] Reche M, Caballero T, López-Trascasa M, Arribas JR, López Serrano MC (2002). Angioedema and transient acquired C1 inhibitor functional deficiency in HIV infection: case report. AIDS.

[8] Macharia WM, Mbaabu C, Wachira L, Muhudhia SO, Moniz G (2005). Ceftriaxone or HIV associated angio-oedema? Case report. East Afr Med J.

[9] Lin RY, Schwartz RA (1993). Cold urticaria and HIV infection. Br J Dermatol.

[10] Fearfield LA, Gazzard B, Bunker CB (1997). Aquagenic urticaria and human immunodeficiency virus infection: treatment with stanozolol. Br J Dermatol.

[11] Karadag AS, Elmas ÖF, Altunay İK (2020). Cutaneous manifestations associated with HIV infections: a great imitator. Clin Dermatol.

[12] Allegra A, Asero R, Giovannetti A, Isola S, Gangemi S (2021). Urticaria and coronavirus infection: a lesson from SARS-CoV-2 pandemic. Eur Ann Allergy Clin Immunol.

[13] Wedi B, Raap U, Kapp A (2004). Chronic urticaria and infections. Curr Opin Allergy Clin Immunol.

[14] Miralles López JC, López Andreu FR, Sánchez-Gascón F, López Rodríguez C, Negro Alvarez JM (2005). Cold urticaria associated with acute serologic toxoplasmosis. Allergol Immunopathol (Madr).

[15] Calado G, Loureiro G, Machado D, Tavares B, Ribeiro C, Pereira C, Luís AS (2012). Streptococcal tonsillitis as a cause of urticaria: tonsillitis and urticaria. Allergol Immunopathol (Madr).

[16] Minciullo PL, Cascio A, Gangemi S (2018). Association between urticaria and nematode infections. Allergy Asthma Proc.

[17] Mirnezami M, Zarinfar N, Sofian M, Botlani Yadegar B, Rahimi H (2020). Mucocutaneous manifestations in HIV-infected patients and their relationship to CD4 lymphocyte counts. Scientifica (Cairo).

[18] Chelidze K, Thomas C, Chang AY, Freeman EE (2019). HIV-related skin disease in the era of antiretroviral therapy: recognition and management. Am J Clin Dermatol.

[19] Cedeno-Laurent F, Gómez-Flores M, Mendez N, Ancer-Rodríguez J, Bryant JL, Gaspari AA, Trujillo JR (2011). New insights into HIV-1-primary skin disorders. J Int AIDS Soc.

[20] Duvic M (1995). Human immunodeficiency virus and the skin: selected controversies. J Invest Dermatol.

